# Redundancy among phospholipase D isoforms in resistance triggered by recognition of the *Pseudomonas syringae* effector AvrRpm1 in *Arabidopsis thaliana*

**DOI:** 10.3389/fpls.2014.00639

**Published:** 2014-11-13

**Authors:** Oskar N. Johansson, Per Fahlberg, Elham Karimi, Anders K. Nilsson, Mats Ellerström, Mats X. Andersson

**Affiliations:** ^1^Department of Biology and Environmental Sciences, University of GothenburgGothenburg, Sweden; ^2^Department of Plant Pathology, Faculty of Agriculture, Tarbiat Modares UniversityTehran, Iran

**Keywords:** phospholipase D, hypersensitive response, *Pseudomonas syringae*, *Arabidopsis thaliana*, phosphatidic acid, pathogen defense

## Abstract

Plants possess a highly sophisticated system for defense against microorganisms. So called MAMP (microbe-associated molecular patterns) triggered immunity (MTI) prevents the majority of non-adapted pathogens from causing disease. Adapted plant pathogens use secreted effector proteins to interfere with such signaling. Recognition of microbial effectors or their activity by plant resistance (R)-proteins triggers a second line of defense resulting in effector triggered immunity (ETI). The latter usually comprises the hypersensitive response (HR) which includes programmed cell death at the site of infection. Phospholipase D (PLD) mediated production of phosphatidic acid (PA) has been linked to both MTI and ETI in plants. Inhibition of PLD activity has been shown to attenuate MTI as well as ETI. In this study, we systematically tested single and double knockouts in all 12 genes encoding PLDs in *Arabidopsis thaliana* for effects on ETI and MTI. No single PLD could be linked to ETI triggered by recognition of effectors secreted by the bacterium *Pseudomonas syringae*. However, repression of PLD dependent PA production by n-butanol strongly inhibited the HR following *Pseudomonas syringae* effector recognition. In addition some *pld* mutants were more sensitive to n-butanol than wild type. Thus, the effect of mutations of PLDs could become detectable, and the corresponding genes can be proposed to be involved in the HR. Only knockout of *PLD*δ caused a loss of MTI-induced cell wall based defense against the non-host powdery mildew *Erysiphe pisi*. This is thus in stark contrast to the involvement of a multitude of PLD isoforms in the HR triggered by AvrRpm1 recognition.

## INTRODUCTION

Plants employ a sophisticated multilayered immune system to defend themselves from pathogenic microbes (Jones and Dangl, 2006; Dodds and Rathjen, 2010). Early defenses are activated upon recognition of conserved molecular patterns of potential pathogens. Recognition of microbe-associated molecular patterns (MAMPs) activates MAMP triggered immunity (MTI) which entails strengthening of the cell wall, transcriptional activation of pathogenesis related (PR) proteins and secretion of low molecular weight antimicrobial substances (Boller and Felix, 2009). MTI is effective against pathogens from several kingdoms and is often sufficient to halt the intruder from colonizing the plant. Microbial co-evolution with plants has provided selective pressure for overcoming MTI and thus increases the possibility to proliferate on or in the plant tissue and cause disease. Adapted pathogens have developed means to suppress MTI. This often comprises the secretion of so called effector proteins, which can interfere with plant defense signaling (Dodds and Rathjen, 2010). In turn, plants have evolved resistance (R) proteins to detect the presence or activity of pathogenic effectors. Recognition of effectors results in a strong and robust defense known as effector triggered immunity (ETI), which often includes the so called hypersensitive response (HR). ETI provides faster, stronger and more specific response to a pathogenic threat than MTI. Though more efficient at stopping adapted pathogens, ETI responses highly overlap those of MTI, the most prominent differential feature being HR leading to localized cell death at the site of infection (Tsuda and Katagiri, 2010). ETI also induces systemic transcriptional reprogramming and defense enhancement through the activation of systemic acquired resistance (SAR; Spoel and Dong, 2012) and long term immunity by epigenetic mechanisms (Molinier et al., 2006; Alvarez et al., 2010).

Phospholipase D (PLD) is a family of enzymes with prominent lipolytic activity in plant tissues that has been recognized for a long time (Hanahan and Chaikoff, 1947; Li et al., 2009). PLD cleaves phospholipids to produce phosphatidic acid (PA) and a free alcohol from the phospholipid head group. The former is known to be a potent second messenger in plants and other organisms (Wang, 2004; Li et al., 2009). PLD and PA dependent signals are implicated in responses to a wide range of abiotic and biotic stresses in higher plants (Laxalt and Munnik, 2002; Li et al., 2009). PA can also be produced by phosphorylation of diacylglycerol (DAG) by DAG kinase (DAGK). DAG in its turn can be produced by phospholipase C (PLC) mediated degradation of phosphoinositides (Wang, 2004). Both pathways are implicated in PA production in response to abiotic as well as biotic stress. PLD dependent PA production can be “inhibited” by primary alcohols, whereas secondary alcohols are inefficient. The effect of primary alcohols is attributable to the preferential use of a primary alcohol for transphosphatidylation by PLD giving rise to an artificial phospholipid rather than PA.

The *Arabidopsis thaliana* (hereafter *Arabidopsis*) genome contains 12 genes encoding PLDs (Li et al., 2009). The PLDs are grouped according to their co-factor requirements and substrate preferences in α, β, γ, δ, ε, and ζ families. The *Arabidopsis* PLDs have roles in responses to various biotic and abiotic stresses. Several of the *Arabidopsis* PLDs have been implicated in responses to abiotic stress (Bargmann and Munnik, 2006): PLDα in drought-, salt- and PLDα, and PLDδ in cold stress (Sang et al., 2001b; Li et al., 2004, 2008; Hong et al., 2008). PLDα has also been implicated in senescence (Fan et al., 1997).

It is well known that PA accumulates in plant cells in response to both MTI and ETI (Bargmann and Munnik, 2006). The relative contribution of PLD and PLC-DAGK to the PA formation during MTI and ETI seems to differ between plant pathogen systems. PLD was previously directly linked to the induction of the HR after recognition of *Pseudomonas syringae* pv. tomato (*Pst*) and *Xanthomonas campestris* effectors (Andersson et al., 2006; Kirik and Mudgett, 2009). PA production is also associated with MTI (van der Luit et al., 2000) and inhibition of PLD was shown to increase the success of a non-adapted powdery mildew in cell wall penetration in *Arabidopsis* (Pinosa et al., 2013).

The individual contribution of different PLD isoforms to plant defense responses is poorly understood. Transcripts of *PLD*α are strongly induced by both virulent and avirulent strains of *Pst* and isoforms of PLDβ are transiently induced by the same strains, whereas PLDγ isoforms are induced only after recognition of the avirulent strain (Zabela et al., 2002). Treatment with the fungal elicitor xylanase as well as both avirulent and virulent strains of *Pst* induce transcriptional activation of PLDβ1 (Laxalt et al., 2001; Zhao et al., 2013). Recently we described how PLDδ is involved in the penetration resistance of *Arabidopsis* against the non-adapted fungal pathogen *Blumeria graminis* Sp *Hordei* (*Bgh*), the causal agent of powdery mildew on barley (Pinosa et al., 2013). The reduced penetration resistance also extended to the more adapted pathogen *Erysiphe pisi* (*Ep*), responsible for the powdery mildew disease of the garden pea. In contrast to the previously described instances where PA generated by PLD seems to act as a positive regulator of plant defense induced by both MTI and ETI, a recent study suggested that PLDβ1 acts like a negative regulator of resistance responses to biotrophic pathogens, HR and salicylic acid dependent defenses in *Arabidopsis* (Zhao et al., 2013).

We herein show that several different *Arabidopsis* PLD isoforms contribute to HR induced by recognition of the *Pst* effector AvrRpm1. On the other hand, cell wall based MTI triggered by the pea powdery mildew *Ep*, which is a non-host pathogen for *Arabidopsis*, is exclusively regulated by a single PLD isoform, PLDδ. To the best of our knowledge, this is the first complete reverse genetics screen of knock outs of all *Arabidopsis* PLD genes for involvement in defense against virulent and avirulent phytopathogenic bacteria.

## MATERIALS AND METHODS

### PLANT MATERIAL

*Arabidopsis* was cultivated on soil in a climate chambers (CLF climatics, Germany) under short day conditions (8 h light/16 h dark, 22°C/18°C, at 120 μmol photons m^-2^ s^-1^ light intensity and 60% relative humidity). The *Arabidopsis rpm1-3* mutant line (Grant et al., 1995) and the *pld* mutant lines (Pinosa et al., 2013) used were all previously described. Garden pea (*Pisum sativum* cv. Kelvedon wonder) was cultivated under green house lights at 22°C.

### ELECTROLYTE LEAKAGE AND BACTERIAL PROLIFERATION ASSAYS

*Pseudomonas syringae* pv. tomato DC3000 strains were maintained on solid *Pseudomonas* agar F (King’s B medium, Biolife, Italy) supplemented with 50 mg l^-1^ rifampicin and 50 mg l^-1^ kanamycin. For electrolyte leakage experiments, exponentially growing cells from overnight plate culturing were suspended in 10 mM MgCl_2_ and diluted to OD_600_ 0.1. The bacterial suspension was vacuum infiltrated into leaf discs (7 mm diameter) of 6–8 week old *Arabidopsis* plants using a SpeedVac vacuum concentrator (Savant, Thermo Electron Corporation, USA). Leaf discs were washed in deionized water and transferred to six well cultivation plates containing 10 mL water (four discs per well). The release of electrolytes from the leaf discs was determined using a conductivity meter (Orion, Thermo scientific) as described (Johansson et al., 2014). In experiments with n- or tert-butanol, the bacteria were suspended in MgCl_2_ solution containing the indicated concentration of n- or tert-butanol, infiltrated and put into culturing plates with 10 mL of deionized water and the same alcohol at the same concentrations.

Bacterial proliferation was measured after syringe infiltration of bacterial suspensions (OD_600_ 0.00002) into the abaxial side of leaves attached to the plant with a needleless syringe. The bacteria were extracted directly or 3 days after infiltration and the number of colony-forming units (CFU) determined after serial dilution and plating as described (Johansson et al., 2014).

To determine the effect of tert- and n-butanol on the growth of *Pst*, exponentially growing cells from overnight culture were re-suspended in 10 mM MgCl_2_ and transferred into liquid cultures of KB media containing n- or tert-butanol at the indicated concentrations. The preparation had an initial optical density of 0.05, corresponding to 2.5^∗^10^7^ CFU^∗^mL^-1^ and were cultivated on a shaker in room temperature for 6 h. An aliquot was taken, serially diluted, plated on KB plates and the number of colonies was determined after 2 days.

### LIPID ANALYSIS

Lipids were extracted from three *Arabidopsis* leaf discs prepared and incubated as above by chloroform methanol extraction as previously described (Andersson et al., 2006) after addition of 0.1 μg of diheptadecanoyl phosphatidylcholine as internal standard. Phosphatidybutanol (PBut) species were analyzed by LC-MS/MS using the chromatographic conditions and instrumental settings previously described (Nilsson et al., 2014) using the MRM transitions described for PBut species (Rainteau et al., 2012). The following molecular species of PBut could be detected: 18:3/18:3, 18:2/18:3, 16:0/18:3, 18:2/18:2, 18:1/18:3, 16:0/18:2, 18:1/18:2, 18:0/18:2. The sum of the mass spectrometric signal for these species divided by that of the internal standard is presented in **Figure 2**.

### *Erysiphe pisi* INOCULATION AND SCORING

The pea powdery mildew fungus *Ep* was maintained on its host plant garden pea. 4 weeks old *Arabidopsis* plants were brush inoculated with *Ep* spores and penetration rate scored at 2 dpi as described (Pinosa et al., 2013) after trypan blue staining (Koch and Slusarenko, 1990). In short, the infection state of at least 50 germinated spores on three separate leaves (3 × 50) was determined by visually inspecting the epidermal surface for stained cells or papillae using a light microscope (100–400 × magnification).

### STATISTICAL ANALYSIS

Statistical analysis was performed as described (Johansson et al., 2014) using GraphPad Prism 6 (GraphPad Software, La Jolla, CA, USA). The final time point (6 h) of ion leakage assays, the penetration rate and the bacterial growth were subjected to one way ANOVA analysis with Tukey’s *post hoc* analysis with *p* < 0.05 considered significant.

## RESULTS

**FIGURE 1:**
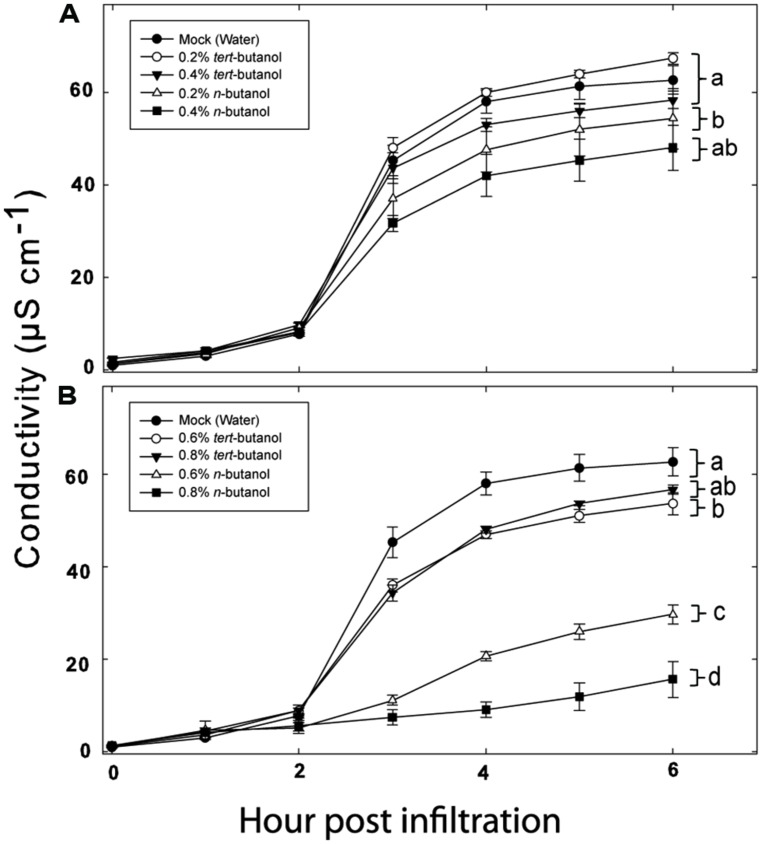
**Phospholipase D (PLD) dependence of hypersensitive response (HR) induction in *Arabidopsis* following recognition of AvrRpm1 expressed by *Pseudomonas syringae* pv. tomato (*Pst).*** Leaf discs were prepared from wild type, Col-0, *Arabidopsis*, infiltrated with *Pst* DC3000:AvrRpm1 suspended in 0.2, 0.4 **(A)**, 0.6 or 0.8 % **(B)** tert- or n-butanol solutions and incubated in deionized water with the same alcohol at the same concentration for 6 h. The conductivity of the bathing solution was measured at the indicated time points. Average of six replicates and SD is shown. Lower case letters represent statistically significant different groups (one way ANOVA, *p* < 0.05) for the 6 h time point. The experiment was performed twice with similar results.

As PLDs are clearly involved in both PTI and ETI, we decided to test the involvement of individual *Arabidopsis* PLD genes on defense responses triggered by recognition of a bacterial effector. The tomato pathovar of *Pseudomonas syringae* DC3000 is normally highly virulent on wild type *Arabidopsis*. However, if the pathogen carries the AvrRpm1 effector gene, the AvrRpm1 protein is recognized by the *Arabidopsis* R-protein RPM1 (Grant et al., 1995). This recognition triggers induction of HR and programmed cell death in the plant. The latter can be measured as loss of electrolytes from leaf tissue into an aqueous solution (Hibberd, 1987; Mackey et al., 2002). To verify the involvement of PLD in HR triggered by AvrRpm1 recognition in *Arabidopsis*, leaf tissue infiltrated with *Pst* DC3000:AvrRpm1 was incubated in solution with different concentrations of n- or tert-butanol and the rate of cell death determined by measuring the electric conductance of the bathing solution (**Figure 1**). The primary alcohol n-butanol is known to inhibit PLD dependent formation of PA, as the alcohol is preferred over water to form an artificial phosphatidylalcohol by transphosphatidylation (Ella et al., 1997). Tert-butanol, on the other hand, is unable to do this. A concentration of 0.6% (v/v) n-butanol caused a decrease in the HR induced by AvrRpm1 recognition by about 40%, whereas 0.8% (v/v) of n-butanol caused an almost complete loss in cell death. Tert-butanol had only a slight effect on the HR as measured by electrolyte leakage, the effect of tert-butanol was apparent only at the two highest concentrations used.

**FIGURE 2:**
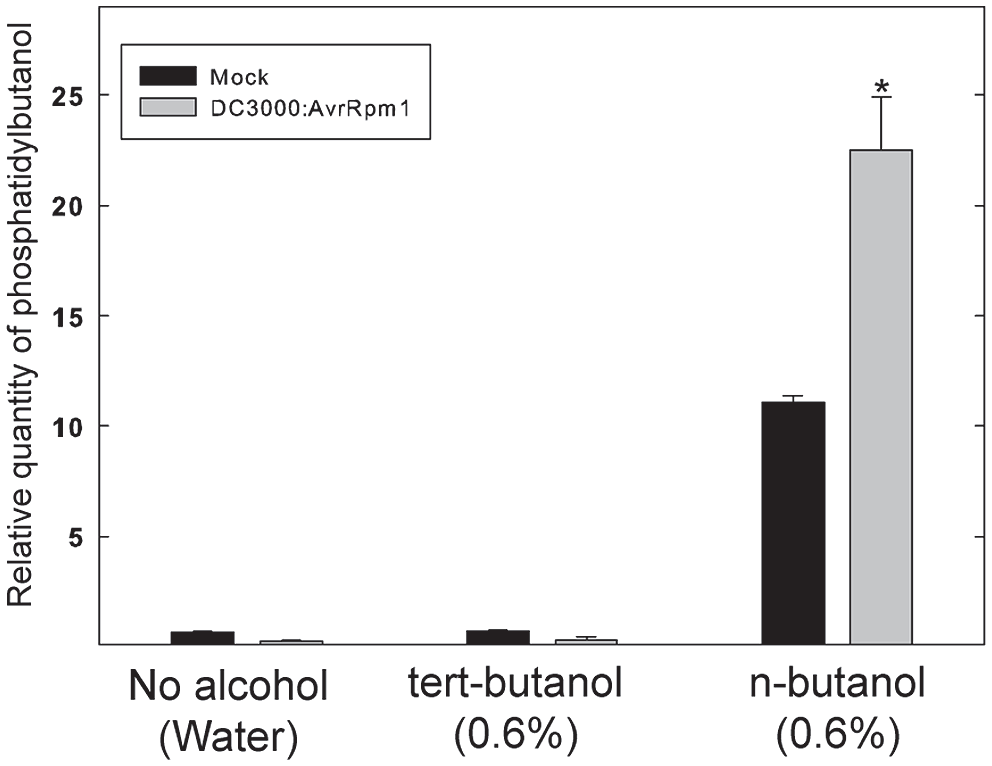
**Formation of Phosphatidybutanol (PBut) in *Arabidopsis* leaf tissue during the HR in the presence of n-butanol.** Leaf discs were prepared from wild type, Col-0, *Arabidopsis*, infiltrated with *Pst* DC3000:AvrRpm1 or MgCl_2_ (mock treatment) in 0.6% tert- or n-butanol water solutions and incubated in deionized water with the same alcohol at the same concentration for 4 h. The lipids were extracted and the amount of PBut formed analyzed by LC-MS/MS. An asterisk indicates a statistically significant (*p* < 0.05, one way ANOVA) difference between mock treatment and infiltration with *Pst* DC3000:AvrRpm1.

To test whether the alcohol by itself had an effect on the bacteria, exponentially growing *Pst* DC3000:AvrRpm1 were incubated with n- or tert-butanol for 6 h and thereafter serially diluted, cultivated on solid medium and the number of colonies determined after 2 days (**Figure S1**). The alcohol treatment caused a significant growth inhibition over 6 h compared to mock treatment. However, there was no significant difference between treatments with tert- or n-butanol. Thus, the effect of n-butanol on the HR is unlikely to be caused by the growth inhibition of the bacteria as the latter did not differ from tert-butanol treatment which did not affect the HR related cell death. To further test that the n-butanol treatment really caused significant formation of PBut during the HR triggered by recognition of AvrRpm1, lipids were extracted from plant tissue treated with tert- or n-butanol 4 h following infiltration with *Pst* DC3000:AvrRpm1 and the amount of PBut formed analyzed by LC-MS/MS (**Figure 2**). There was a clear induction of PBut formation following recognition of AvrRpm1 in the presence of n-butanol at the same (0.6%) concentration that caused a significant decrease in HR related cell death. This thus confirms activation of PLD during the HR and formation of PBut in the presence on n-butanol. These results taken together support previously published data (Andersson et al., 2006; Kirik and Mudgett, 2009) that the HR induced by recognition of bacterial effectors in *Arabidopsis* is strongly dependent on formation of PA by PLD.

### A HIGH DEGREE OF REDUNDANCY AMONG PLD ISOFORMS IN THE ETI SIGNALING

**FIGURE 3:**
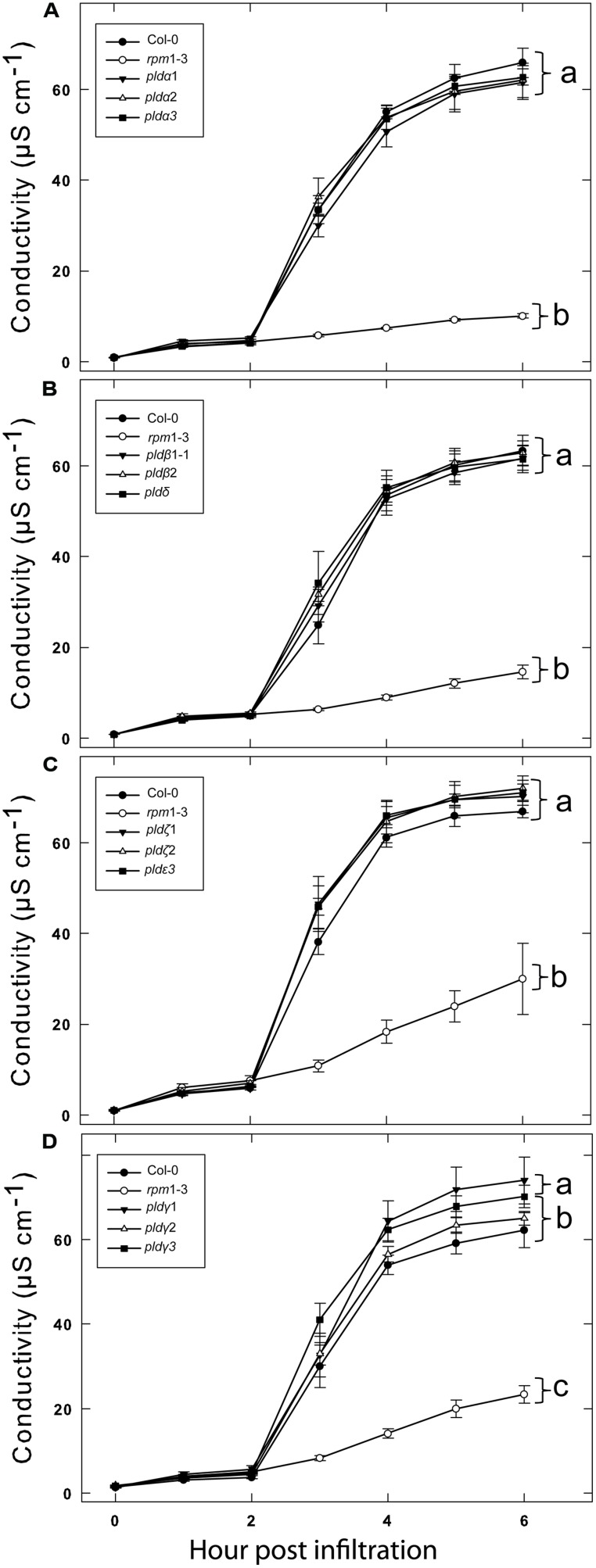
**A high degree of redundancy in PLD genes in *Arabidopsis* involved in the HR in induced by AvrRpm1 recognition.** Leaf discs were prepared from the indicated lines, infiltrated with *Pst* DC3000:AvrRpm1 and incubated in deionized water. Loss of cellular electrolytes was measured as the conductance of the bathing solution at the indicated time points. Col-0 and *rpm1-3* are included in all experiments **(A–D)** together with the indicated subset of PLD knock out mutants. Average of six replicates and SD is shown. Lower case letters represent statistically significant different groups (one way ANOVA, *p* < 0.05) for the 6 h time point. The experiment was performed twice with similar results.

**FIGURE 4:**
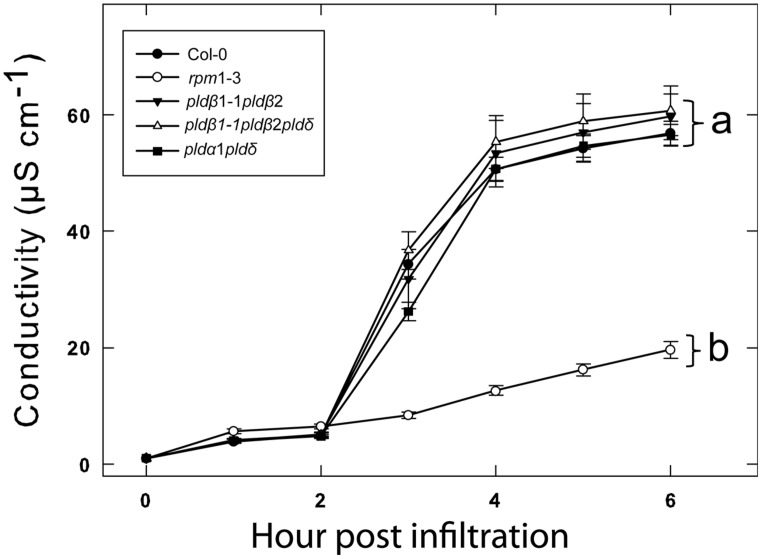
**Double knockout mutants of PLDα, δ, and β confers no additional loss of HR following AvrRpm1 recognition.** Leaf discs were prepared from the indicated lines, infiltrated with *Pst* DC3000:AvrRpm1 and incubated in deionized water. The loss of cellular electrolytes was measured as the conductance of the bathing solution at the indicated time points. Average of six replicates and SD is shown. Lower case letters represent statistically significant different groups (one way ANOVA, *p* < 0.05) for the 6 h time point. The experiment was performed twice with similar results.

We previously tested a panel of PLD mutants for effects on cell wall based resistance to barley powdery mildew and found that PLDδ was involved in the MAMP triggered signaling involved in the defense reaction (Pinosa et al., 2013). However, as effector triggered resistance differs significantly from MAMP triggered defense responses, we tested if the PLD-mediated effect on could be attributed to any particular of the 12 PLD genes in the *Arabidopsis* genome. Single (**Figure 3**), double and triple (**Figure 4**) *pld* T-DNA mutants (Pinosa et al., 2013) were assayed for HR induced after infiltration with *Pst* DC3000:AvrRpm1. This revealed no clear reduction in HR induced ion leakage for any of the tested mutants compared to wild type. The *pld*γ*1* and *pld*γ*3* mutants appeared to demonstrate a slightly elevated cell death response following AvrRpm1 recognition (**Figure 3D**). Taken together, this suggests that there is a high degree of genetic redundancy among the PLD isoforms activated during HR induced by AvrRpm1 recognition.

**FIGURE 5:**
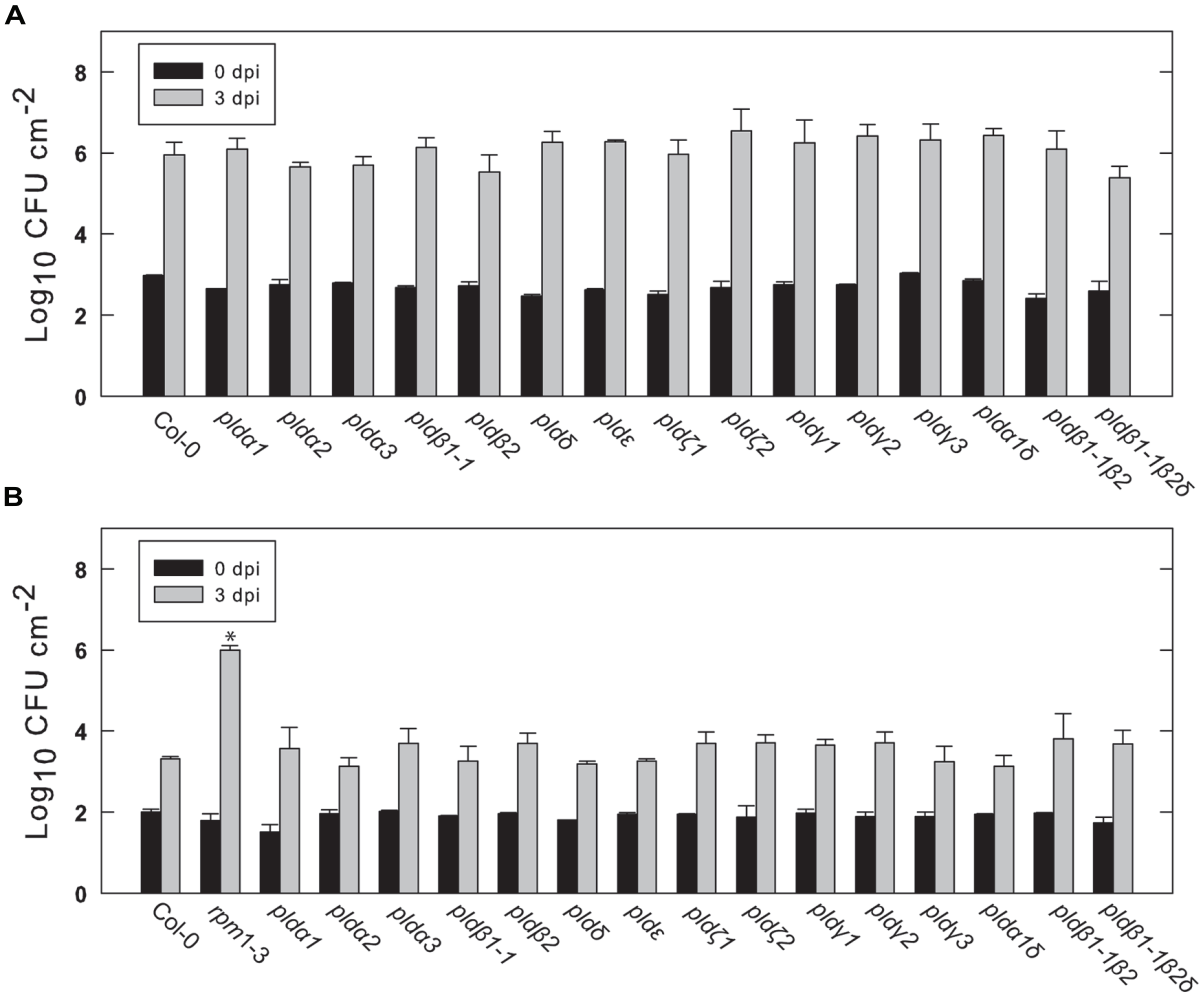
**Growth of virulent and avirulent *Pst* in various PLD knockout mutants.** *Arabidopsis* leaves attached to the plant were infiltrated with a suspension of *Pst* DC3000 **(A)** or DC3000:AvrRpm1 **(B)**. The bacteria in the leaves were extracted immediately or after 3 days and quantified by serial dilution and cultivation on solid medium. Average and SD of three replicates is shown. An asterisk indicates statistically significant differences from wild type (Col-0) at 3 dpi. The experiments shown were repeated twice with similar results.

We next tested the different *Arabidopsis* lines for their ability to restrict growth of the virulent *Pst* DC3000 and the avirulent strain DC3000:AvrRpm1. As expected, over a period of 3 days DC3000 multiplied in wild type leaves by about a thousand times (**Figure 5A**). The growth of DC3000 was not significantly affected in any of the tested mutant lines. The avirulent strain DC3000:AvrRpm1 grew about 10-fold in 2 days in wild type Col-0 and this was not significantly affected in any of the tested mutants (**Figure 5B**). The *rpm1-3* mutant, which is unable to recognize AvrRpm1, demonstrated bacterial multiplication by about 10000 times. To conclude, none of the tested PLD single, double or triple mutants demonstrated any apparent change in resistance toward virulent and avirulent *Pst* DC3000.

### HR PHENOTYPES OF *pld* MUTANTS IN COMBINATION WITH INHIBITION OF PLD DEPENDENT PA FORMATION

**FIGURE 6:**
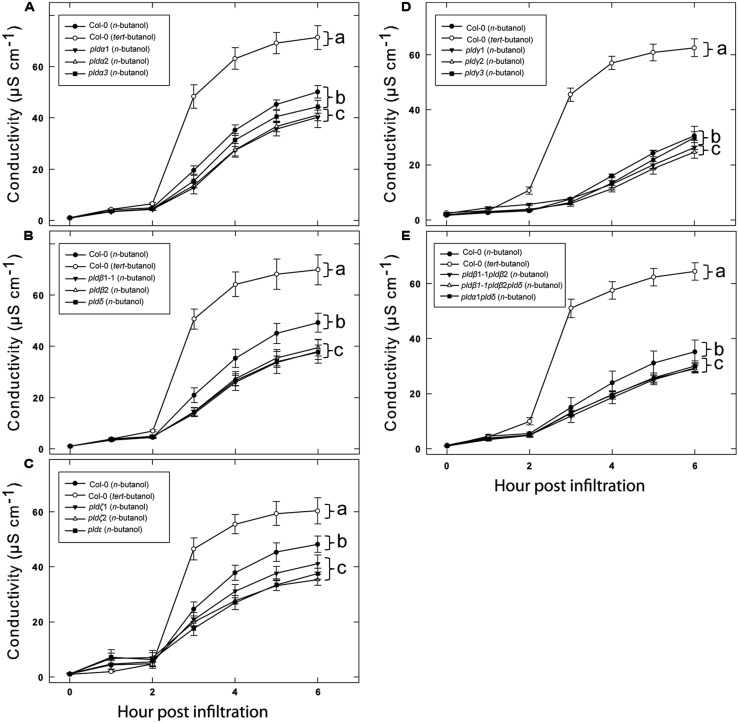
**Additative effects of n-butanol and loss of single PLD genes on HR cell death following recognition of AvrRpm1.** Leaf discs were prepared from the indicated lines, infiltrated with *Pst* DC3000:AvrRpm1 in 0.6% tert- or n-butanol as indicated and incubated in deionized water with the same alcohol. Col-0 treated with tert- and n-butanol is included in all experiments **(A–E)** together with the indicated subset of PLD knock out mutants. The loss of cellular electrolytes was measured as the conductance of the bathing solution at the indicated time points. Average of six replicates and SD is shown. Lower case letters represent statistically significant different groups (one way ANOVA, *p* < 0.05) for the 6 h time point. The experiment was performed twice with similar results.

While none of the tested *pld* mutants displayed a clear reduction in effector induced HR, the involvement of PLDs in this defense reaction was apparent as treatment with n-butanol clearly affected the plants ability to mount HR and formation of PBut in connection with this was observed. We thus reasoned that the PLD activity in response to AvrRpm1 recognition is likely caused by the activation of several PLD isoforms and that the individual contributions might be so small that the single knock outs show no phenotype. Thus, if the overall activity of PLD is lowered by addition of n-butanol, it might be possible to detect the effect of loss of single PLD isoforms. To test this, wild type (Col-0) and all the *pld* mutants were infiltrated with 0.6% n-butanol together with *Pst* DC3000:AvrRpm1 (**Figure 6**). As a control, the wild type was also treated with 0.6% tert-butanol. As expected, 0.6% n-butanol caused a significant reduction in ion leakage following AvrRpm1 recognition compared to treatment with 0.6% tert-butanol in wild type. The single mutants *pld*α*1*, *pld*α*2, pld*β*1, pld*β*2, pld*δ, *pld*ζ*1, pld*ζ*2,* and *pld*ε all displayed 10–20% statistically significant reductions in HR compared to wild type when treated with 0.6% n-butanol (**Figures 6A–C**). The mutants *pld*γ*1* and *pld*γ*2* also displayed a statistically significant reduction in ion leakage induced after AvrRpm1 recognition, this effect was however smaller than for the other mutants (**Figure 6D**). Finally, the double mutants *pld*β*1 pld*β*2* and *pld*α*1* *pld*δ, as well as the triple mutant *pld*β*1 pld*β*2 pld*δ, displayed a similar conditional reduction of ion leakage as shown for the single mutants (**Figure 6E**).

### PLDδ IS THE ONLY PLD ISOFORM INVOLVED IN TRIGGERING CELL WALL BASED DEFENSE AGAINST A NON-HOST POWDERY MILDEW

**FIGURE 7:**
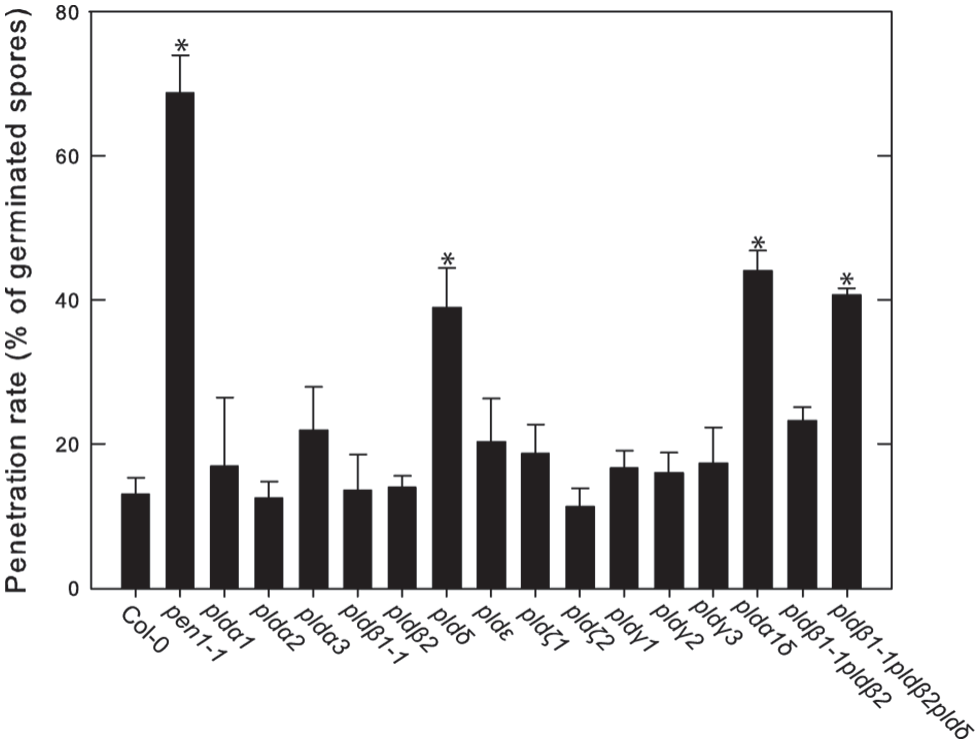
**Involvement of PLDδ in microbe-associated molecular pattern (MAMP) triggered cell wall based defense against pea powdery mildew.** The indicated wild type (Col-0) and mutant lines were inoculated with *Ep* spores and trypan blue stained at 2 dpi. The rate of successful penetration of the epidermal cell wall was calculated from counting of at least 50 germinated spores on three independent leaves. Average and SD is shown. An asterisk indicates statistically significant difference from wild type (Col-0), *p* < 0.05, one way ANOVA.

We previously reported that PLDδ was the only isoform involved in PLD-dependent cell wall based defense against the non-host powdery mildew *Bgh* and that the *pld*δ mutant also demonstrated a loss of penetration resistance toward pea powdery mildew *Ep* (Pinosa et al., 2013). We thus tested the response of the full panel of PLD mutants to *Ep* (**Figure 7**). To this end plants were inoculated with *Ep,* leaves stained with trypan blue 2 days post infection and scored for disease progress. The *pen1-1* mutant was included as a control as it has a severely deficient cell wall based resistance response against non-host powdery mildews (Collins et al., 2003). The number of germinated spores that successfully penetrated the epidermal cell wall was about 15% in wild type (Col-0), whereas *pen1-1* allowed about 70% of the germinated spores to penetrate the epidermal cell wall. Among the tested PLD single mutants, only *pld δ* displayed any increase in penetration rate compared to wild type. Higher order mutants containing the *pld*δ displayed the same phenotype as the single mutant. There was no change in frequency of single epidermal cell death following successful penetration in any of the tested mutants compared to wild type.

## DISCUSSION

The HR was described a century ago, only recently has the molecular details of the process from the recognition of pathogenic effectors to the “auto destruction” of the host cell begun to be elucidated (Mur et al., 2008). PLDs have been shown to play an important role in the induction of HR following recognition of pathogenic effectors. This was previously demonstrated using a system where the *Pst* effector AvrRpm1 was expressed *in planta* after the selective inhibition of PLD dependent production of PA by primary alcohols (Andersson et al., 2006). We herein show that the same holds true for effector delivered by live *Pst* bacteria infiltrated into the tissue. Alcohol treatment caused a reduction in growth of *Pst* cultured *in vitro*. However, as the growth inhibition effect did not differ between tert- and n-butanol, and since n-butanol had a strong effect on the HR, we deem it most likely that this non-specific effect of the alcohol is not the primary cause of the inhibition of the HR related cell death. Furthermore, as formation of PBut could be observed in tissue after inoculation with *Pst* DC3000:AvrRpm1 in the presence of n-butanol, it is likely that the effect of n-butanol was primarily exerted on PA production by PLD.

Phosphatidic acid has been shown to directly cause oxidative damage and cell death when infiltrated into plant tissue (Sang et al., 2001a; Park et al., 2004; Andersson et al., 2006). However, the contribution of PLD derived signals needed for the induction of HR and programmed cell death seems to vary between different effectors. HR induced by recognition of AvrRpm1 is highly PLD dependent, but is also inhibited by inhibition of PLC (Andersson et al., 2006). It was thus proposed that PLD activation is dependent of PLC activity in the case of AvrRpm1 triggered HR. On the other hand HR induced by AvrRpt2 is only inhibited if both PLD and PLC activity are affected at the same time and AvrBsT is of intermediate sensitivity to PLD inhibition (Kirik and Mudgett, 2009). This highlights that different effector recognition events trigger slightly different intracellular signaling pathways. Even though many components may be shared between the different effector response pathways, the extent to which specific signal transducers are involved in the responses appears to vary.

Of the 12 different PLDs encoded by the *Arabidopsis* genome no single gene knockout led to a decrease in HR induced after recognition of AvrRpm1. Since chemical inhibition of PLD dependent PA formation by primary alcohols strongly affects the HR, this point to a high degree of redundancy among the PLD genes in induction of HR following effector recognition. MTI, on the other hand, as tested here and previously (Pinosa et al., 2013) was found to be affected by the loss of a single gene, PLDδ. This difference is fully compatible with the notion that ETI is characterized by robust and redundant activation of intracellular signaling, whereas signaling leading to MTI is associated with a lower degree of redundancy (Sato et al., 2010). The lack of discernable HR phenotype of the tested PLD knockouts was also reflected in that none of the tested mutants displayed any difference in ability to restrict growth of *Pst* expressing AvrRpm1.

When combined with partial inhibition of the HR by n-butanol induced transphosphatidylation, several of the single knockout mutants revealed a decreased HR after recognition of AvrRpm1. Specifically, *pld*α*1*, *pld*α*2*, *pld*β*1*, *pld*β*2*, *pld*δ, *pld*ζ*1*, *pld*ζ*2,* and *pld*ε all displayed a conditional HR phenotype in the presence of n-butanol. We interpret this as that multiple PLDs are activated and contribute to PA production which stimulates the HR induced by AvrRpm1 recognition in *Arabidopsis*. However, alternative explanations exist such as that certain PLDs become more active in the absence of other isoforms. This regulation could be both at a transcriptional and/or at a post translational level. A small decrease in HR following AvrRpm1 recognition was previously reported for the double mutant *pldα1 pld δ*. This finding was reported in a doctoral thesis, but never formally published in a journal (http://dare.uva.nl/record/281626). Although we found no phenotype of the double mutant, it can easily be envisioned that a small phenotype might sometimes be present as these two PLDs represent the most abundant PLD transcripts in *Arabidopsis*. Taken together, our data points to that the small contributions of many PLDs together provide enough PA to form an active signal. A likely activation mechanism for the multiple PLDs is the very strong and sustained increase in cytosolic calcium observed to follow recognition of bacterial effectors (Grant et al., 2000).

We found no evidence of that the *pld*β*1* would contribute to increased HR in response to AvrRpm1 recognition as reported for recognition of AvrRpt2 (Zhao et al., 2013). This could be due to differences between the signaling pathways induced by different effector types. The *pen3* mutant for example demonstrates different phenotypes depending on whether it is treated with *Pst* expressing AvrRpm1 or AvrRpt2 (Kobae et al., 2006; Johansson et al., 2014). The previously reported strongly decreased proliferation in leaf tissue of virulent *Pst* DC3000 in the *pld*β*1* mutant (Zhao et al., 2013) was not apparent in our hands. There are differences in the experimental setup such as light intensity and density of the bacterial inoculum which could influence the outcome. It should however be noted that the *pld*β1-1 line used herein is identical to the T-DNA line used in the study by Zhao et al. (2013). Further studies are needed to clarify this point and further investigate among other factors the effect of different bacterial titers on the defense reaction and the phenotype of the mutant.

To conclude, we herein report that at least eight different PLD isoforms in *Arabidopsis* contribute to signaling in HR triggered by AvrRpm1 recognition. In contrast, loss of just one of the major PLD isoforms is sufficient to significantly affect MTI dependent defense responses.

## Conflict of Interest Statement

The authors declare that the research was conducted in the absence of any commercial or financial relationships that could be construed as a potential conflict of interest.

## SUPPLEMENTARY MATERIAL

Figure S1 **Effect of tert- and n-butanol on the growth rate of Pseudomonas syringae *in vitro*.** An overnight culture of *Pst* was diluted and transferred into liquid cultures with the respective concentration of butanol, grown for 6 h on shaker, serial diluted and plated on KB plates. The number of colonies was determined after 2 days incubation in room temperature. Shown are averages and SD of three replicates. An asterisk indicates statistically significant difference from water, *p* < 0.05, one way ANOVA. The experiment was performed twice with the same conclusion.
