# Supplementary figures and images for: Redundancy among phospholipase D isoforms in resistance triggered by recognition of the Pseudomonas syringae effector AvrRpm1 in Arabidopsis thaliana
Source: Front Plant Sci. 2014 Nov 13;5:639. doi: 10.3389/fpls.2014.00639 (PMC4230166; doi:10.3389/fpls.2014.00639)

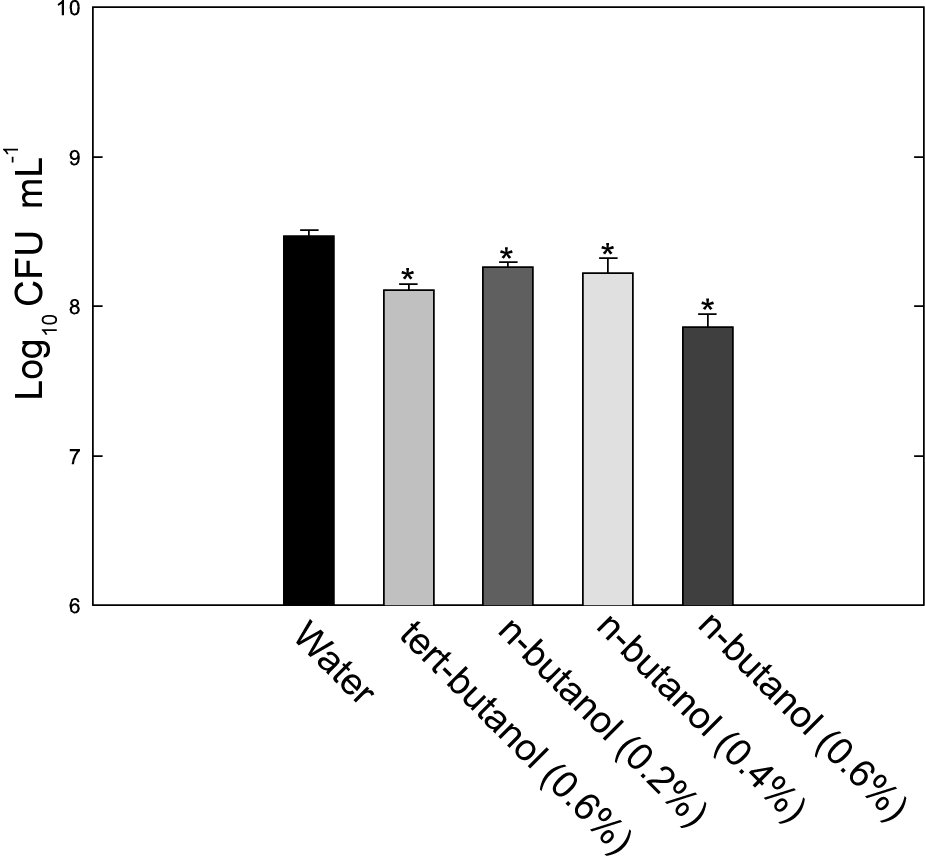

Supplement: Figure S1 — Effect of tert- and n-butanol on the growth rate of Pseudomonas syringae in vitro. An overnight culture of Pst was diluted and transferred into liquid cultures with the respective concentration of butanol, grown for 6 h on shaker, serial diluted and plated on KB plates. The number of colonies was determined after 2 days incubation in room temperature. Shown are averages and SD of three replicates. An asterisk indicates statistically significant difference from water, p < 0.05, one way ANOVA. The experiment was performed twice with the same conclusion. [file Image_1.TIF]
